# The time course of person perception from voices in the brain

**DOI:** 10.1073/pnas.2318361121

**Published:** 2024-06-18

**Authors:** Nadine Lavan, Paula Rinke, Mathias Scharinger

**Affiliations:** ^a^Department of Biological and Experimental Psychology, School of Biological and Behavioural Sciences, Queen Mary University of London, London E1 4NS, United Kingdom; ^b^Research Group Phonetics, Institute of German Linguistics, Philipps-University Marburg, Marburg 35037, Germany; ^c^Research Center “Deutscher Sprachatlas”, Philipps-University Marburg, Marburg 35037, Germany; ^d^Center for Mind, Brain & Behavior, Universities of Marburg & Gießen, Marburg 35032, Germany

**Keywords:** voice, first impressions, representations, EEG, trustworthiness

## Abstract

When listeners hear a voice, they rapidly form a complex first impression of who the person behind that voice might be. We characterize how these multivariate first impressions from voices emerge over time across different levels of abstraction using electroencephalography and representational similarity analysis. We find that for eight perceived physical (gender, age, and health), trait (attractiveness, dominance, and trustworthiness), and social characteristics (educatedness and professionalism), representations emerge early (~80 ms after stimulus onset), with voice acoustics contributing to those representations between ~100 ms and 400 ms. While impressions of person characteristics are highly correlated, we can find evidence for highly abstracted, independent representations of individual person characteristics. These abstracted representationse merge gradually over time. That is, representations of physical characteristics (age, gender) arise early (from ~120 ms), while representations of some trait and social characteristics emerge later (~360 ms onward). The findings align with recent theoretical models and shed light on the computations underpinning person perception from voices.

## Significance

Within a second of exposure to a voice, listeners make up their mind about what kind of person they are dealing with. While often not fully accurate, these first impressions can guide our social interactions and affect how we behave toward other people. When forming these first impressions from voices, listeners do not perceive individual person characteristics independently of one another but tend to get a complex, seemingly holistic impression of a person. In the current study, we used electroencephalography (EEG) to trace how listeners put together these complex impressions by tracking whether, when, and how different individual person characteristics can be perceived.

Listening to natural sounds creates rich perceptual experiences, during which we rapidly process, categorize, and evaluate the different auditory signals in our environments. Human voices are one such socially salient auditory signal. When we hear a voice, we hear a person such that listeners readily form complex impressions of that person behind the voice (1–3). These first impressions can be formed based on less than a second of exposure to an unfamiliar person’s voice. First impressions from voices and faces have been shown to guide and inform our behavior such that voice and face properties have been linked to who people vote for in an election (4–7), whether a landlord decides to rent a property to a person (8), who people want to affiliate with (9), and how harshly criminals are sentenced in court (10, 11). It is therefore surprising that it is not yet well understood how we form and assemble these complex first impressions from voices. Do we perceive some person characteristics more quickly than others, or are all characteristics perceived holistically at the same time? Are some characteristics emergent from combinations of other characteristics? At which point—if at any point—do perceived person characteristics become abstracted and invariant to acoustic voice properties?

Behavioral studies suggest a staggered time course of person perception from voices, with the perception of physical characteristics, such as age and gender, requiring as little as 25 ms of exposure to a voice (12, 13), the perception of trait characteristics, such as dominance and trustworthiness, requiring 400 ms of exposure, (12, 14, 15) and the perception of social characteristics, such as level of education and “poshness”, requiring 800 ms of exposure (12). While these studies suggest that different amounts of voice information are necessary to perceive different person characteristics and form first impressions, they can only speak to postperceptual judgments. The studies therefore do not directly speak to when and how impressions themselves first emerge while listening to a voice.

To examine how person perception from voices unfolds over time, from stimulus onset onward, previous voice perception studies have used electroencephalography (EEG). In contrast to behavioral studies, EEG measures continuous neural responses to voices, without requiring an explicit behavioral response. This then makes it possible to measure when complex impressions first emerge by tracking neural responses. These EEG studies show that gender (16), attractiveness (17, 18), expressed traits such as confidence (19–21) and politeness (22, 23), and person identities (24–26) can all be differentiated in different components of event-related potentials evoked within a few hundred milliseconds following stimulus onset.

In line with hierarchical models of voice perception (2, 27–30), electrophysiological studies of person perception from voices often interpret early effects in the evoked responses to voices to reflect the processing of basic acoustic properties (~100 ms, 29), which is then followed by the processing of acoustic properties that are perceptually salient for a specific person characteristic (e.g., F0 and formant frequencies for gender perception, ~200 ms), and finally by higher-order processing and evaluation of the perceptual information (~400 ms). Empirical studies have indeed shown that gender perception becomes invariant to differences in voice fundamental frequency (F0; correlated with perceived pitch) by 170 ms after stimulus onset (16). Similarly, experiments show that early (90 ms to 230 ms after onset) but not later representations of face identities (from 460 ms after onset) can be partially explained by image similarity (31). Furthermore, converging evidence for sound category perception (32) (human voices vs. animal calls, vs. auditory scenes) shows that early neural responses (up to 150 ms) are best explained by acoustic stimulus properties, while later responses (from 200 ms onward) are better explained by abstracted category-level information. However, these EEG studies have tended to only examine the perception of a single person characteristic at a time, with different types of stimuli, tasks, analysis methods, and experimental designs being used across studies. Thus, while each study is informative in itself, it can be difficult to draw more wide-ranging conclusions across these heterogeneous studies about how different person characteristics are perceived from the voice over time in relation to one another.

Recent work on face perception has explored when and how information about multiple person characteristics from faces are represented and thus processed in the brain, using Representational Similarity Analysis (RSA). Dobs and colleagues (33) examined how face perception unfolds over time for a number of person characteristics (age, gender, identity, identity familiarity) within the same study. The authors report that representations of age can be detected earlier than gender (after 60 ms vs. 72 ms respectively), followed by representations of identity (91 ms), and identity familiarity (402 ms). Furthermore, Ambrus and colleagues (31) also report that gender affects representations of face identities during an early time window (~150 ms after stimulus onset). Together, these studies show that person-related information from faces is processed rapidly and that this rapid processing unfolds over time.

In the current study, we provide a report of how multivariate person perception from voices unfolds over time and ask which information shapes representations of person characteristics across different processing stages. While previous research on face perception (31, 33) has primarily modeled person perception based on objectively quantifiable characteristics (e.g., age perception being tied to a person’s true age), our study takes a different approach, where we model person perception via purely subjective impressions formed by listeners. By moving from modeling “objective” characteristics to subjective impressions, we can both conceptually refocus person perception research on perception per se (as opposed to, e.g., signal detection) and can also broaden the scope of our study to also examine characteristics for which objective measures are difficult to obtain (e.g., trustworthiness, dominance).

As a basic prediction, we expected that representations of person characteristics could be decoded from the neural data. We furthermore predicted that representations of the different person characteristics could be linked to voice acoustics early in the brain’s evoked response, with later representations being invariant to voice acoustics (16, 31, 32). Finally, we predicted that person perception from voices unfolds over time, where primarily physical characteristics [e.g., sex, age, health) should be represented earlier than more trait or social characteristics (e.g., educatedness, professionalism, trustworthiness, (1, 12, 33)].

## Results

**Fig. 1.**
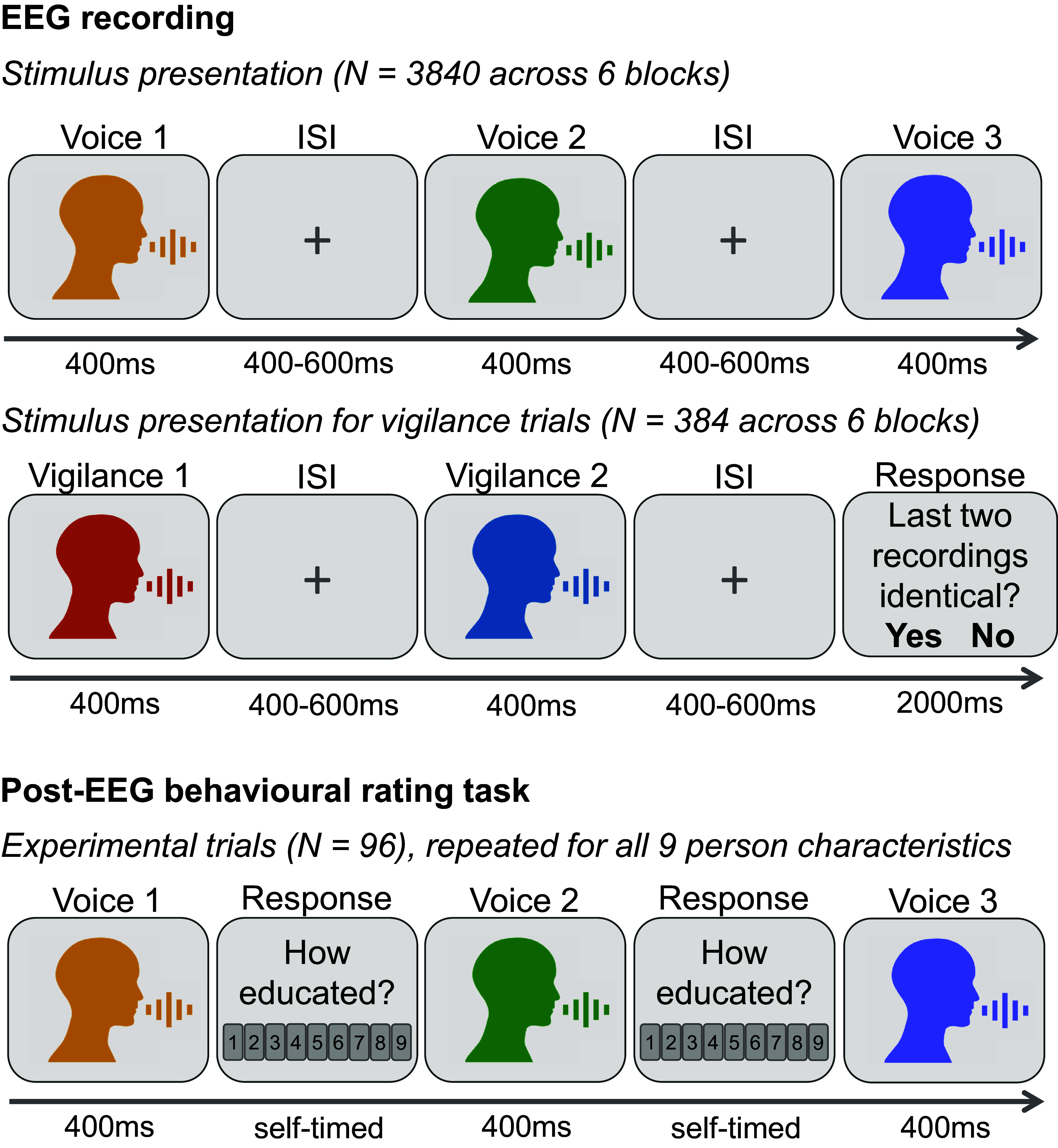
Illustration of the experimental procedure and trial types for the EEG recording session and behavioral rating task. Please note that EEG trials were epoched for the analysis such that they started 100 ms before stimulus onset and ended for the shortest ISIs 300 ms after stimulus offset (or 700 ms after stimulus onset). There was therefore no temporal overlap been trials as they were epoched for analysis.

In the current study, we analyzed EEG and behavioral ratings data collected across two testing sessions from 32 participants. During the EEG session, participants were presented with 96 voice recordings, comprising different 32 voices producing three spoken vowels each (/a/, /i/, /u/; stimulus duration = 400 ms). We chose to include vowels in our study in order to focus on how person perception is achieved based on information conveyed in the voice quality or sound of a voice (as opposed to, for example, what words are said). Participants monitored the voice recordings in a 1-back vigilance task for whether the two previously heard sounds had been identical (i.e., a repeated presentation) or whether they had been two different recordings (i.e., differing in vowel and/or speaker; see Fig. 1). During the behavioral testing session, participants listened to all voices from the EEG session again and now provided subjective ratings for physical, trait, and social person characteristics (specifically: gender, age, health, attractiveness, dominance, trustworthiness, educatedness, and professionalism). We then used RSA to link the EEG data to behavioral ratings and track when and how first impressions are represented in the brain. Before reporting on the RSA, we will first briefly examine the behavioral ratings and describe a neural time course for when listeners can distinguish between different sounds, thus outlining the time window during which we can expect to find representations of person characteristics in the EEG data.

### Perceptual Characteristics of the Voice Recordings.

**Fig. 2.**
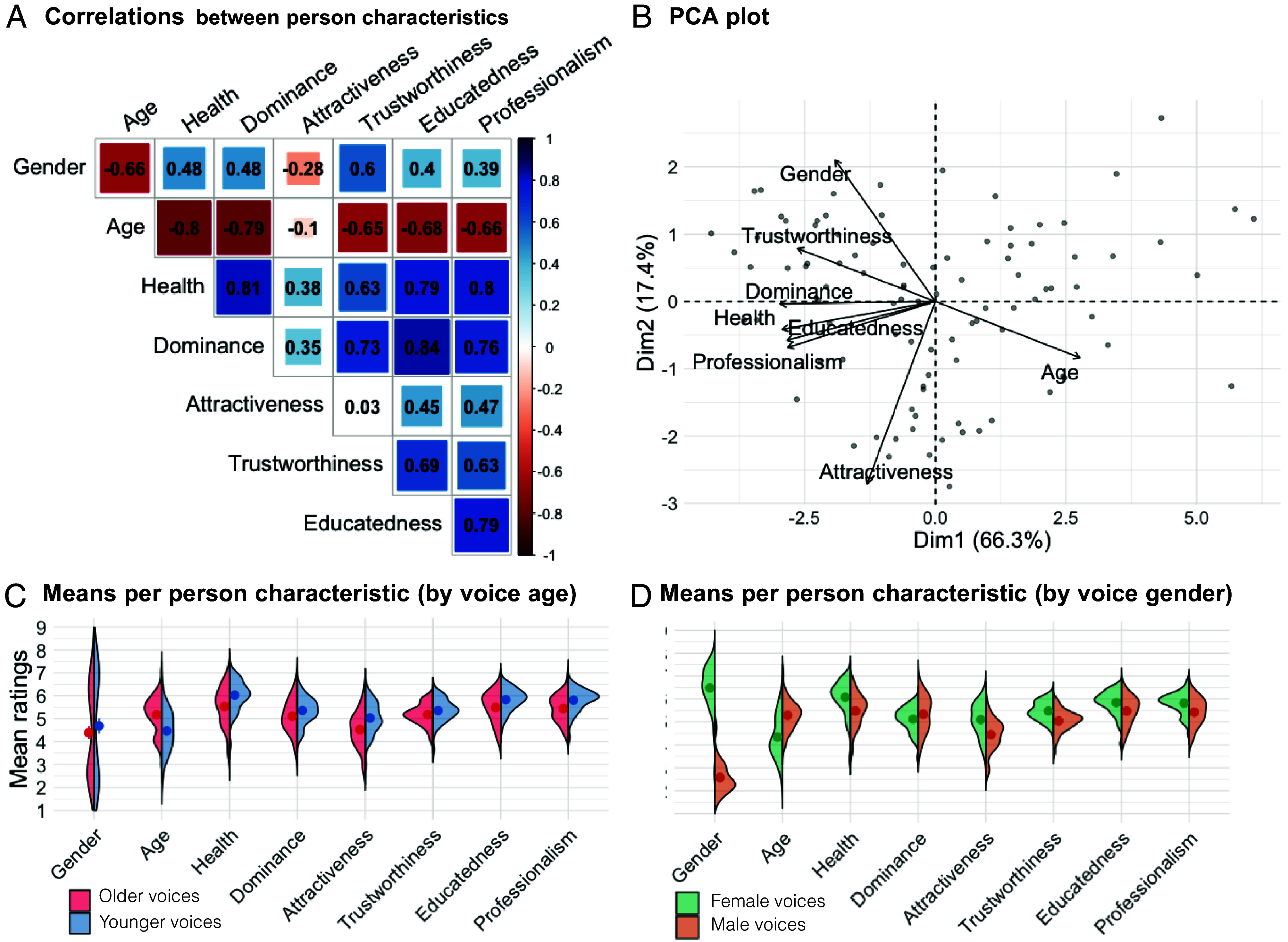
(*A*) Matrix of Spearman’s correlations between mean behavioral ratings for all person characteristics. (*B*) PCA plot showing the loading of person characteristics and individual voices on the first two components of the PCA (with oblimin rotation). (*C*) Violin plots of item-wise mean ratings for each person characteristic, split by objective voice age as reported in the demographics of the Saarbrücker Voice Database. Dots show means for younger vs. older voices, respectively, and half violins show density plots to illustrate the data distributions. (*D*) Violin plots of item-wise mean ratings for each person characteristic, split by objective voice gender as reported in the demographics of the Saarbrücker Voice Database.

Behavioral evaluations of the different person characteristics are highly intercorrelated with one another (Fig. 2*A*). Intercorrelations such as the ones observed here are well documented in the voice and face perception literature (12, 34, 35) and have been explained in light of “halo” or overgeneralization effects. Halo effects describe how perceivers tend to extend the percept of one positively or negatively valence person characteristic to others of similar valence (e.g., considering an attractive person to also be trustworthy, (36–39)). Beyond such cognitive biases to categorize people along valenced characteristics, some correlations in our data may also be explained by world knowledge: Health and age ratings are negatively correlated, perhaps reflecting that older people are indeed often less healthy than young people. Further, given that person-related information, such as identity, is in some cases less readily perceived from voices than from faces, high intercorrelations could also be explained by listeners having not particularly well-formed or individuated representations of the different characteristics (35).

**Fig. 3.**
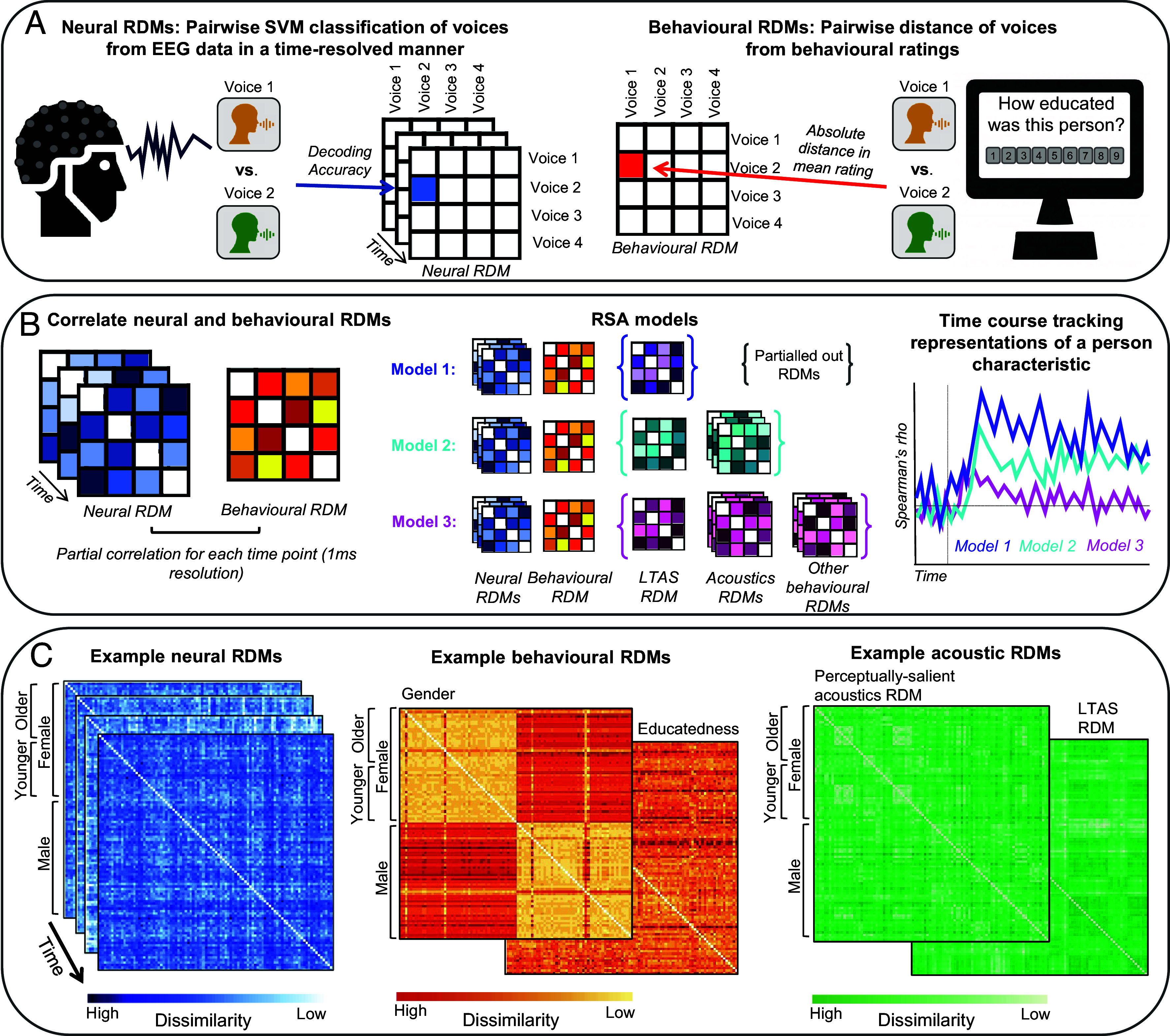
Summary of the analysis approach for the RSA. (*A*) As a basis for the RSA, we created RDMs including pairwise dissimilarity measures derived from the neural and behavioral data. (*B*) Neural and behavioral RDMs were then related to one another via partial correlations. We ran three different models, with each model indexing increasingly abstracted representations of person characteristics. By running the partial correlations for each time point, a timeline of when representations of different person characteristics can be decoded from the neural data can then be established. (*C*) Example neural, behavioral, and acoustic RDMs.

In light of the high correlations between person characteristics, we conducted a principal component analysis (PCA with oblimin rotation) for the person characteristics on the mean ratings per voice recording. Dimension reduction techniques such as PCA have previously been used in social psychology and the voice and face perception literature to abstract low-dimensional “trait spaces” from complex, intercorrelated ratings (14, 34, 40–44). While the reported dimensions differ somewhat between existing studies, they tend to report that trait perception can be described along dimensions of trustworthiness (or warmth), dominance (or competence), and sometimes attractiveness/youthfulness. Similarly, for the current data, two components with eigenvalues over 1 were identified, explaining 83.7% of the variance in the data. Trustworthiness and dominance both load on Component 1 alongside health, educatedness, and professionalism. Gender and attractiveness load highly on Component 2 (Fig. 3*B*). While we replicate that intercorrelated person characteristics can be mapped into low-dimensional space, there are thus some differences between the current study and previous research (14, 41, 43, 44) since trustworthiness and dominance are load highly on the same PC in our dataset, which is unexpected in relation to previous findings (14, 44). This unexpected feature may be idiosyncratic to our stimulus set and/or may be explained by the number and choice of person characteristics included in the current study. Most previous work only included (many more) trait characteristics while excluding, e.g., physical properties, which may have affected the PCA results. In general, however, the correlation analysis and PCA, however, further underline that different person characteristics are not perceived independently of one another but are highly interdependent.

### Tracking the Time Course of Representations of Sound Decoding in the Brain.

To track the time course of person perception, we ran a time-resolved RSA on the EEG and behavioral data (Fig. 3). We computed the averaged EEG responses (epoched to −100 ms to 700 ms in relation to stimulus onset, with there being no overlap in time between epoched EEG responses, even for the shortest ISIs) to each individual voice recording across repetitions and electrodes for each participant. For each time point, we computed the dissimilarity of the EEG data for each pair of voices via a fivefold cross-validated decoding accuracy using support vector machines (SVMs). This resulted in one 96 * 96 neural representational dissimilarity matrix (RDM) of decoding accuracies for each participant and time point (33, 45).

#### Different voice recordings can be distinguished in the brain within 66 ms of hearing a voice.

**Fig. 4.**
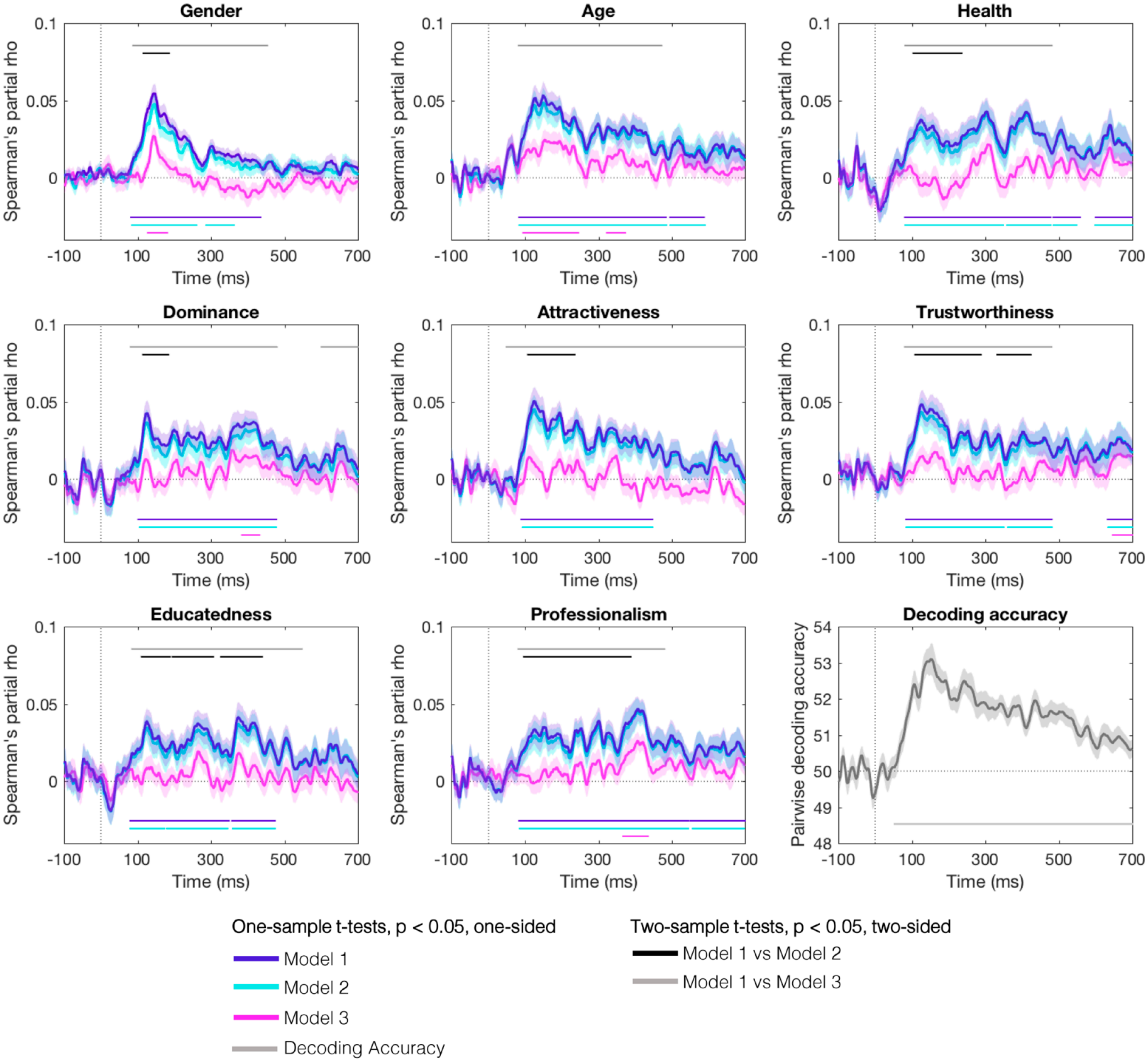
Time course of person perception from voices, plotted for each person characteristic. In the first eight plots, purple lines show time course of person perception derived from Model 1 (partial correlations between neural and the relevant behavioral matrix, with the LTAS matrix partialled out). Aquamarine lines show time course of person perception derived from Model 2 (partial correlations between neural and the relevant behavioral matrix, with the LTAS matrix and matrices capturing perceptually salient voice acoustics partialled out). Pink lines show time course of person perception derived from Model 3 (partial correlations between neural and the relevant behavioral matrix, with the LTAS matrix and matrices capturing perceptually salient voice acoustics, plus matrices of all other known person characteristics partialled out). The final panel showed the average pairwise decoding accuracy, indexing when individual voice recordings can be decoded from the neural data. Shaded areas show the SEM; significance is marked for *P* < 0.05 (one-sided) for one-sample *t* tests and *P* < 0.05 (two-sided) for two-sample *t* tests.

We first examined neural voice decoding to determine at which points the EEG data discriminate between the different voice recordings. For this purpose, we averaged all pairwise decoding accuracies for each time point in the upper triangle (excluding the diagonal) of the neural RDM for all participants. This analysis revealed significant neural voice decoding between 66 ms and 700 after stimulus onset, with a peak after 154 ms (53.1% mean decoding accuracy, t(31) = 7.15, Cohen’s *d* = 1.23, Fig. 4, gray line). The neural RDMs therefore encode information that distinguishes between pairs of voice recordings from within 66 ms of hearing a voice. This time window thus delineates when we can expect to find evidence for representations of different person characteristics in the RSA. This time course of voice decoding is similar to time courses that have been reported for general sound decoding (32, 46), the time courses reported for the decoding of visual stimuli (33, 47), as well as the time courses of neural responses to famous faces established via electrocorticographical recordings (48).

### Tracking the Time Course of Representations of Person Characteristics in the Brain.

To track when representations of the different person characteristics can be detected, we related the EEG data to the behavioral ratings. We computed behavioral RDMs based on the ratings data collected from participants after the EEG session. These behavioral RDMs index the absolute distances of all pairs of voices in relation to their mean rating on the physical, trait, and social characteristics investigated. We furthermore computed acoustic RDMs to examine when representations of person characteristics become invariant to acoustic differences.

We used the neural, behavioral, and acoustic RDMs to compute Spearman’s partial rank correlations between the lower triangles (excluding the diagonal) of combinations of the different matrices for each time point. Specifically, we ran three models (Fig. 3*B*):

Model 1 is our “baseline” model, in which we track when different person characteristics are represented in the brain, only partialling out low-level acoustic properties. Specifically, we compute the time-resolved correlations between behavioral and neural RDMs partialling out a matrix of the pairwise dissimilarity of the long-term average spectrum (LTAS) of the voice recordings. This LTAS matrix accounts for overarching differences in the spectral shape of voice recordings, thus accounting for, e.g., differences in vowel category among other low-level acoustic properties.

Model 2 examines representations of person characteristics, after accounting for perceptually salient acoustic differences in the sounds of voices. To do this, we computed the time-resolved correlations between behavioral and neural RDMs partialling out 1) a matrix of the pairwise dissimilarity of the LTAS and 2) matrices of the absolute differences between 4 principal components characterizing the perceptually salient acoustic properties of the voice recordings. Comparing Models 1 and 2 can therefore outline when representations of person characteristics become invariant to (perceptually salient) acoustic information.

Model 3 examines whether and when any abstracted representations of person characteristics can be detected that are independent of both voice acoustics and all other (known) perceived person characteristics of the voices. To do this, we computed the time-resolved correlations between behavioral and neural RDMs partialling out 1) a matrix of the pairwise dissimilarity of the LTAS, 2) perceptual-salient acoustics, and 3) all other averaged behavioral matrices.

These models therefore characterize representations of person characteristics of increasing abstraction along a processing hierarchy (27–29), first only accounting for low-level acoustics (Model 1), adding perceptually salient voice acoustics (Model 2), and finally also accounting for any other known higher-order perceptual properties (Model 3). For further details, see the Methods section below. For an analysis including “Model 0,” where no information is partialled out, see *SI Appendix, Supplementary Analysis 2*.

#### Model 1: Representations of physical, trait, and social person characteristics can be decoded from within 100 ms of hearing a voice.

To address our main research question, we asked at which points in time representations of the different person characteristics could be found in the neural data, after accounting for low-level acoustic stimulus properties (e.g., differences between different vowel pronunciations). As indicated by significant correlations between neural and behavioral RDMs, representations for all person characteristics were found from 80 ms to 102 ms after stimulus onset onward. These representations could be detected until at least 435 ms after stimulus onset for gender but often for longer, with significant clusters even stretching up to the end of the sampled time window for professionalism (see Fig. 4, purple lines and Table 1 for details). Against our initial predictions, Model 1 provides no evidence for a staggered time course of person perception from voices, where some representations emerge earlier than others. There are, however, some differences in how long representations can be decoded: Representations of gender, dominance, attractiveness, and educatedness cannot be detected beyond shortly after stimulus offset, while representations for the remaining person characteristics remain detectable for longer.

**Table 1.** Time points for significant clusters and peaks where representations of different person characteristics could be decoded from the neural data

Model	Person characteristic	Time points of significant cluster(s)	Time points of peak
Model 1	Gender	82 ms to 435 ms	145 ms
	Age	83 ms to 588 ms	149 ms
	Health	81 ms to 558 ms; 600 ms to 700 ms	305 ms
	Dominance	102 ms to 478 ms	125 ms
	Attractiveness	89 ms to 447 ms	125 ms
	Trustworthiness	84 ms to 481 ms; 632 ms to 700 ms	127 ms
	Educatedness	80 ms to 474 ms	374 ms
	Professionalism	83 ms to 700 ms	407 ms
Model 2	Gender	85 ms to 260 ms; 287 ms to 362 ms;	147 ms
	Age	84 ms to 482 ms; 496 ms to 590 ms	149 ms
	Health	82 ms to 348 ms; 359 ms to 478 ms; 486 ms to 548 ms; 599 ms to 700 ms	303 ms
	Dominance	106 ms to 477 ms	123 ms
	Attractiveness	93 ms to 447 ms	125 ms
	Trustworthiness	85 ms to 351 ms; 362 ms to 481 ms; 635 ms to 700 ms	125 ms
	Educatedness	80 ms to 173 ms; 180 ms to 345 ms; 359 ms to 474 ms	374 ms
	Professionalism	85 ms to 545 ms; 557 ms to 697 ms	407 ms
Model 3	Gender	127 ms to 181 ms	144 ms
	Age	94 ms to 245 ms; 323 ms to 373 ms	154 ms
	Health	–	
	Dominance	384 ms to 432 ms	358 ms
	Attractiveness	–	
	Trustworthiness	647 ms to 700 ms	163 ms
	Educatedness	–	
	Professionalism	367 ms to 535 ms	407 ms

Significance is defined as *P* < 0.05 (one-sided) for one-sample *t* tests.

For all person characteristics, a first peak can be seen between 100 ms and 200 ms (Fig. 4). This first peak is also the highest peak for most person characteristics. Only for health, educatedness, and professionalism do the highest peaks occur later (Table 1). These findings help may characterize some of the descriptive differences in the nature of the time courses across different person characteristics: Representations for health, educatedness, and professionalism (as indexed by correlation strength) are more stable and consistent across time, while representations for the remaining person characteristics show a steady decrease in correlation strength across time.

#### Model 2: Perceptually salient voice acoustics are linked to the early stages of person perception from voices.

Models of voice perception propose that the processing of perceptually salient acoustic information takes place early on during voice processing, while higher-order perceptual information, such as identity, speech, or emotion, is processed later (2, 27, 28, 30, 39). Evidence from face and natural sound perception confirms this proposal, where studies show that visual or acoustic stimulus properties are encoded in representations of faces or natural sounds during early time points but that representations become invariant to stimulus properties later on (31, 32, 46).

We tested this proposal in Model 2, where we characterized how perceptually salient acoustic information shapes and affects the time course of person perception from voices. To this end, we reran the analysis outlined for Model 1, now additionally partialling out perceptually salient acoustic properties in addition to the low-level acoustics. Model 2 shows that representations that are invariant to perceptually salient voice acoustics for all person characteristics can be found along relatively similar time course as were observed in Model 1 (see Fig. 4 aquamarine lines, Table 1 for details). The only notable change in when representations can be detected can be seen for gender perception, where representations can only be detected between 85 ms and 362 ms in Model 2 (instead of 82 ms to 435 ms in Model 1).

This is, however, not to say that acoustic information does not contribute to and shape neural representations of person characteristics. As predicted, we observe that perceptually salient voice acoustics consistently affect the early parts of the time courses of person perception when comparing the timelines of Model 1 and Model 2 (Table 2): Correlations are significantly lower for Model 2 compared to Model 1 between 96 ms and 236 ms after stimulus onset for gender, health, dominance, and attractiveness. For trustworthiness, educatedness, and professionalism the effects of voice acoustics are even more prolonged, stretching until 388 ms and beyond. No differences in correlations were found between Model 1 and Model 2 for representations of age, although similar nonsignificant trends are apparent in a cluster ranging from 124 ms to 182 ms (see Fig. 4; *Z* value for cluster = 1.88, *Z* threshold for significance = 1.96).

**Table 2.** Time points for differences in the time course between Model 1 and Model 2 and Model 1 and Model 3

Comparison	Person characteristic	Time points of significant cluster(s)
Model 1 vs. Model 2	Gender	104 ms to 186 ms
	Age	-
	Health	104 ms to 236 ms;
	Dominance	115 ms to 184 ms
	Attractiveness	107 ms to 225 ms
	Trustworthiness	109 ms to 288 ms; 332 ms to 424 ms
	Educatedness	111 ms to 189 ms; 195 ms to 306 ms; 327 ms to 439 ms
	Professionalism	96 ms to 388 ms
Model 1 vs. Model 3	Gender	88 ms to 453 ms
	Age	82 ms to 472 ms
	Health	82 ms to 482 ms
	Dominance	81 ms to 479 ms; 600 ms to 700 ms
	Attractiveness	49 ms to 700 ms
	Trustworthiness	81 ms to 481 ms
	Educatedness	85 ms to 547 ms
	Professionalism	81 ms to 481 ms

Significance is defined as *P* < 0.05 (two-sided) for two-sample *t* tests.

Finding early contributions of perceptually salient voice acoustics is in line with predictions from hierarchical models of voice perception (2, 27–30). In the current study, the modeled voice acoustics only partially explain the variance in the EEG data such that there is still evidence representations of person characteristics, as indicated by correlations of Model 2 remaining significantly above 0 for all characteristics from ~ 80 ms to at least 435 ms.

#### Model 3: Independent, abstracted representations of different person characteristics emerge at different time points.

In Model 3, we finally tested whether and when abstracted representations of person characteristics could be detected that are independent of both voice acoustics and all other (known) perceived person characteristics of the voices. Any representations identified in Model 3 can therefore be considered to be highly abstracted, reflecting variance in the data that is unique to the specific person characteristic in question. Perhaps unsurprisingly given the substantial intercorrelations between the behavioral evaluations of the person characteristics, most evidence for representations disappears in Model 3: Two-sample *t* tests confirm that there are significant differences between Model 1 and Model 3 across most of the sampled time window, with correlations being consistently and considerably lower for Model 3, (see Fig. 4, pink lines; and Table 2). Furthermore, one-sample *t* tests against 0 show that no significant representations of health, attractiveness, and educatedness remain detectable. This could therefore suggest that these characteristics are entirely captured by or may be entirely emergent from combinations of other characteristics and aligns with the observation in the behavioral ratings that impressions of almost all person characteristics are highly intercorrelated (see Fig. 2*A*). Intriguingly, however, abstracted representations of gender (127 ms to 181 ms) and age (94 ms to 373 ms) remain detectable earlier in the time while evidence comes later for independent abstracted representations of dominance (384 ms to 432 ms), trustworthiness (647 ms to 700 ms), and professionalism (367 ms to 535 ms). These findings align with our prediction that representations of physical characteristics emerge earlier than representations of some of the trait and social person characteristics. It is furthermore intuitive that these differences between types of characteristics only arise when examining highly abstracted representations: Since all person characteristics are highly correlated, any differences between time course for individual characteristics will have been obscured by these correlations in previous models. Differences between individual person characteristics can therefore only emerge when examining different person characteristics independently from other characteristics.

## Conclusions

We characterize the time course of person perception from voices in the brain, reporting evidence that representations for a range of physical, trait, and social person characteristics can be found from ~80 ms after voice onset. Our findings furthermore provide empirical support for proposals from models of voice perception (2, 27–30), showing that early representations are partially shaped by voice acoustics, while somewhat later representations are invariant to acoustic information. These findings speak to there being different processing stages transforming acoustics-based representations to highly abstracted representations of person characteristics, which had to date mainly been discussed on a theoretical level for person identity perception from voices (27–29). We furthermore find that person characteristics are highly intercorrelated such that there is only relatively sparse evidence for independent and abstracted representations of individual person characteristics. Where abstracted representations that are also independent of all other known person characteristics can be detected, representations emerge early for the physical characteristics, gender and age, and later for the trait and social characteristics, dominance, trustworthiness, and professionalism. These findings can thus speak to person perception from voices occurring in a staggered manner, unfolding over time, aligning with previous behavioral work for voices (12) and work from the face perception literature (33). The lack of independent abstracted representations for some person characteristics alongside the staggered emergence for others, in turn, raise intriguing possibilities about how complex first impressions arise: Our findings could suggest that person characteristics that are available to perception earlier in time may influence and partially guide how person characteristics that may become available somewhat later in time may be perceived (2, 12). Similarly, finding no evidence of abstracted representations for some person characteristics (here: health, attractiveness, educatedness) may suggest that these characteristics are fully dependent on or emergent from representations of other person characteristics.

Finding such a hierarchy of different person characteristics during person perception might offer a mechanistic explanation for how overgeneralization and halo effects might arise (36–39), leading to the perception of person characteristics being intercorrelated. If, for example, an early impression of “old age” would facilitate the formation of impressions for certain person characteristics but not others (e.g., “less dominant” rather than “more dominant”), codependencies may arise resulting in clusters of person characteristics that are usually perceived in concert (2). This interpretation of our findings is, however, highly speculative, and future research is required to firmly establish whether such causal hierarchies do or do not exist.

The current study reports some very promising initial findings, although much more work is required to gain a thorough understanding of how listeners perceive other people from voices. Specifically, human voices are much more variable in naturalistic settings than we have modeled in the current study such that voices in naturalistic settings are thus also much richer in information. For example, information encoded in what a person is saying (i.e., the words they use) and how a person chooses to say things (i.e., the speaking style, register, and accent they use) undoubtedly contribute to person perception from voices. Crucially, however, the impressions of other people derived from the voice quality as we investigated it in the current study are likely to be among the very first information listeners can glean from a voice from very little exposure to a voice (12, 15, 33). Linguistic and paralinguistic information, in contrast, usually unfolds in the voice over several seconds, with even individual syllables of words lasting several hundred milliseconds in themselves. Focusing on voice quality only and thus only looking at very first impressions was ideal for the main research question we posed: How do listeners arrive at a (first) complex impression of a person based on very little exposure to a voice? However, future work needs to examine how the different types of information routinely encoded in a voice are used and integrated to form a complex impression of a person. It is, for example, a largely open question whether the more slowly emerging linguistic and paralinguistic information in a voice would add to—or, in parts, override—a first impression based on the sound of someone’s voice as soon as it becomes available within the first few seconds of listening to a person.

While the current study focused on how the very first and earliest complex impressions are formed, we can also broaden our time window of interest to look beyond first impressions that include our initial appraisal of a person’s characteristics based on their voice quality and linguistic and paralinguistic information. While first impressions have been shown to have real-life impact (4–8, 10, 11, 18), they are likely fleeting and will change over time, as listeners accumulate more information about a person. To date, it is largely unclear how listeners dynamically update their first impression with additional information as it becomes available to move to a second impression that gets established over the course of either a longer interaction or several short interactions. Similarly, we do not know when and how listeners eventually transition over the course of several interactions from dynamic first and second impressions of (relatively) unfamiliar people to having established a stable impression of a now-familiar person.

Recent work in speech and language comprehension uses continuous exposure to spoken narratives to model how listeners use and integrate different levels of information (e.g., acoustic, phonetic, morphological, syntactic, semantic) over time to process these complex auditory stimuli and extract the meaning of words, sentences, and whole stories from the auditory signal (49–52). Building on the initial findings around the time course of person perception in voices from this paper, it might eventually be possible to leverage the methods and experimental designs from the speech and language processing literature to model how first impressions of a person emerge, get updated in light of additional information, and stabilize over the course of several minutes of exposure to a voice. Once we can model how impressions are dynamically formed over time, it may then be possible to more directly measure whether and how the impressions influence behavior and thus model how vocal (and) information shapes social interactions.

## Methods

### Participants.

In total, 35 participants were tested in this experiment. 3 participants were excluded from all analyses due to technical issues. This resulted in a final dataset of 32 participants (mean age = 25.22 y, SD = 4.17 y, 21 female). All participants were native German speakers and had no self-reported hearing difficulties. Written informed consent was obtained from all before participating in the experiment. The study was approved by the Ethics Board of the German Linguistic Society (DGfS) and was in accordance with the Declarations of Helsinki.

### Materials.

#### Experimental stimuli.

We selected 96 recordings of sustained vowels from the Saarbrücker Voice Database (53). The current study thus examines person perception from voices based largely on information encoded in the acoustic/perceptual quality of the voice, thus limiting the role of linguistic information. Further, since we predicted a staggered time course of when different person characteristics would be perceived from voices, our stimuli needed to be quasi-steady state, meaning that any segment of the recording should be relatively similar to any other segment from the same recording. Vowels obey this requirement, meaning that they convey similar amounts of person-related information throughout their duration (12, 15). Had we used more naturalistic spoken word stimuli (e.g., “Hello”), the spoken words would have been differentially informative at different points in time with regard to person characteristics (e.g., the voiceless fricative /h/ does not include pitch information while the subsequent vowel /ə/ does). This would have as a result confounded our experimental design, making any temporal effects difficult to interpret. Finally, vowels are frequently used in electrophysiological studies targeting early auditory evoked potentials (AEPs). This is due to their acoustic property of being quasi-harmonic sounds, to which AEPs are particularly sensitive (54–56).

Within the 96 voice recordings, there were 32 individual speakers, with each speaker being represented by recordings of three vowels (/a/, /i/, /u/). Half of the speakers were female, the other half were male. Furthermore, half the stimuli were sampled from young adults (20 to 34 y), and the other half from older adults (50 to 74 y). Age ranges for female and male voices were matched. This sampling strategy was implemented to introduce variability in the basic demographics within the stimulus set. Each voice recording was cropped to 400 ms in duration with a linear fade in and fade out of 25 ms. All voice recordings were rms normalized for intensity using Praat (57).

#### Vigilance stimuli.

We selected an additional 24 voice recordings to be used as the vigilance stimuli. These vigilance stimuli were also selected and processed from the Saarbrücker Voice Database (53) and sampled in the same way as the experimental stimuli. The vigilance stimuli thus comprised intensity-normalized voice recordings of 400 ms from eight speakers (half female, half younger adults), which were represented by recordings of three vowels each.

### Procedure.

#### EEG recording.

Continuous EEG was recorded from 32 electrodes, using an active electrode system (Brain Products), in combination with a BrainAmp amplifier, connected to an ActiCAP control box. Electrodes were attached to a nylon cap (EasyCap) and arranged according to the 10 to 20 system (58). The reference electrode was attached to the nose (59, 60). The ground electrode was placed on the forehead between the channels Fp1 and Fp2. The recording was performed with the BrainVision Recorder 1.21.0303 (Brain Products Inc., Gilching, Germany) using a sampling rate of 1 kHz and an online filter between 0.1 and 1,000 Hz.

Participants were seated in an acoustically and electrically shielded room at a distance of approximately 1 m from a computer screen. During the EEG recording, participants were presented with voice recordings and instructed to listen carefully to these voice recordings Voice recordings were presented via loudspeakers placed on both sides of the computer screen and were delivered at a comfortable volume that was kept the same for all participants (approximating an intensity level of 70 dB). While listening to the voice recordings, participants completed a 1-back vigilance task: For ~9% of all trials, participants were prompted on the computer screen to report whether the two previously presented sounds had been identical (i.e., a repeated presentation) or whether they had been two different recordings (i.e., differing in vowel and/or speaker; see Fig. 1). Vigilance trials were spaced evenly within each block, never occurring at the start of a block nor immediately following a previous vigilance trial. Vigilance data from two participants were not recorded due to a technical error. However, there were no indications that these participants were not paying attention (e.g., participants were observed to respond to vigilance trials during the EEG recording and provided high-quality behavioral data). As a result, their data were included. Accuracy for the vigilance task was over 90% correct for all other participants such that no participants were excluded based on inattention during the EEG session.

In total, participants were presented with 40 repetitions of each of the 96 voice recordings with an additional 16 repetitions of the 24 vigilance trials. For vigilance trials with pairs of identical recordings, a vigilance stimulus was presented twice. For the pairs of nonidentical recordings, a vigilance stimulus was presented once, preceded by a (random) test stimulus. Thus, there were 3,840 experimental trials plus 384 vigilance trials (which included presentations of 576 vigilance stimuli in total across the experiment. Trials were spread evenly across 6 blocks, including 704 trials each. Stimulus onsets were jittered between 800 ms and 1,000 ms relative to the previous stimulus onset. Given the 400 ms of stimulus duration, silent interstimulus intervals (ISIs) were therefore between 400 ms and 600 ms in duration. Each block lasted around 12 min.

#### Behavioral ratings.

In a separate testing session following the EEG recording, participants rated all 96 voice recordings for 9 person characteristics—gender, age, health, attractiveness, dominance, trustworthiness, educatedness, professionalism, and sexual orientation—on a scale from 1 (“not at all [characteristic]”) to 9 (“very [characteristic],” with the exceptions of gender, age, and sexual orientation, where the scales ranged from “very feminine”/”like a young adult/not heterosexual at all” to “very masculine”/”like an old adult”/”very heterosexual” (Fig. 1).

The selected set of person characteristics was chosen based on an experiment that collected free descriptions of people from voices (1, 12). Across different descriptions of physical and social descriptions, the characteristics included here (gender, age, health, educatedness, and professionalism) were among the most frequently mentioned person characteristics listed by lay listeners, marking them as salient during person perception from voices. The remaining characteristics were selected based on existing literature identifying attractiveness, dominance, and trustworthiness as being among the fundamental characteristics for trait perception (14, 34, 40–44, 61). Three additional rating scales examining the perceptual properties of vowel stimuli (“brightness,” “openness,” and “prototypicality”) were included, which are part of a separate project and not reported here. These 12 perceptual rating scales were blocked, and the order of blocks was counterbalanced across participants. The rating task was self-paced, and the order of stimuli within blocks was also randomized. The behavioral rating task was implemented in Gorilla Experiment Builder (62).

#### Acoustic measures.

To characterize the acoustic properties of the voice recordings, we first extracted the LTAS from each voice recording using PRAAT (57). The LTAS is a measure that can account for any overarching differences in the frequency spectrum between voice recordings. We furthermore extracted the acoustic measures described in a psychoacoustic model of voice quality (14). These acoustic measures have been shown to be perceptually salient in the context of voice quality perception. Specifically, we extracted the F0 mean, and the mean of the first 4 formants (F1; F2; F3; and F4) alongside a measure of formant dispersion (DF, calculated using the formula by Reby and McComb (2003). Additionally, we characterized the spectral shape of the harmonic source (H1-H2, H2-H4; H4-H2kHz; H2kHz-H5kHz) as well as the inharmonic source (cepstral peak prominence, Energy). All measures were extracted using VoiceSauce (using Praat to measure F0 and formants). We additionally included a measure of the harmonics-to-noise ratio (HNR, extracted in PRAAT) since HNR has been highlighted as also being perceptually salient in the context of voice perception (63).

### Data Analysis.

#### EEG preprocessing.

Raw EEG data were preprocessed with fieldtrip (58) and with custom Matlab scripts (Matlab version 2021a, Mathworks, Inc., Natick, MA, USA). Electrophysiological responses were analyzed in time windows from –100 ms prestimulus onset to 700 ms poststimulus onset. Epochs were defined based on stimulus durations (400 ms) and based on the typical stimulus onset asynchrony (SOA) range of experiments with foci on auditory evoked components.

Erroneous EEG channels were identified using the joint probability approach with a threshold of 4 SD in the frequency domain (64). Channels marked as erroneous were then interpolated using spherical spline interpolation (65). At most, 2 channels had to be interpolated per participant (mean = 0.66 channels per participant, <2% of electrodes within our dataset). Subsequently, after 100 Hz low-pass filtering, EEG data underwent an artifact analysis within fieldtrip. This involved detecting muscle artifacts as well as epochs with amplitudes exceeding 150 µV (peak-to-peak). Automatic artifact detection led to the exclusion of individual trials, resulting in the rejection of between 1.83 and 9.14% of all trials per participant (mean: 5.12%). Following this, cleaned data were band-pass filtered between 0.1 and 30 Hz, using a 4th order zero-phase Butterworth filter (following suggestions in ref. 66) and a baseline correction was applied. Baseline correction involved the subtraction of the averaged 100 ms prestimulus voltage from the epochs. Furthermore, data were rereferenced against the temporal electrodes closest to the preauricular landmarks (Tp9 and Tp10), approximating a linked mastoid reference.

Electro-ocular (EOG) artifacts were identified by an independent component analysis (ICA) applied to the epoched data described above. Component decomposition was carried out within fieldtrip (using the fastICA algorithm). Components resembling electro-occulogram (EOG) activity based on visual inspection and illustrated in ref. 67 were considered artifacts. For an analysis of Auditory Evoked Components, please see Supplementary Analysis 1.

#### Preprocessing of behavioural ratings.

For the behavioral ratings, we first screened the data to exclude the data from any participants who gave the same response for more than 80% of all trials for ratings of a given characteristic. As a result, one participant’s data were excluded from professionalism ratings, another participant’s data for gender, sexual orientation, and trustworthiness ratings, and another participant’s data for sexual orientation and trustworthiness ratings. We then computed the intraclass correlation coefficient (ICC) to assess interrater agreement. Specifically, we computed a two-way random model (ICC2k). Agreement was moderate to high (range = 0.63 to 0.99) for all person characteristics, apart from sexual orientation, where agreement was poor (0.33). We therefore refrained from further analyzing the data for sexual orientation.

#### RSA.

We conducted RSA to link the EEG data to the behavioral and acoustic data. For the EEG data, we first subaveraged all trials of each individual voice recording by randomly assigning each trial to one of five splits and averaging the trials in each split. These splits were then randomly divided into test and training sets via a hold-one-out approach (i.e., 1 split for test vs. 4 splits for training). We applied multivariate noise normalization to the test data based on the training data using the Epoch method (45). Following this, we quantified the neural dissimilarity between all 9,216 pairs of voice recordings (i.e., 96 voices * 96 voices) via linear classification analysis using SVMs Libsvm in MATLAB (33, 45) for each time point at a resolution of 1 ms (from −100 ms to 700 ms relative to stimulus onset, matching the temporal resolution of the EEG recording). This linear classification analysis resulted in a decoding accuracy value (chance performance = 50%) for each pair of recordings. The subaveraging procedure was repeated for 50 permutations. Each pairwise decoding accuracy for each time point was averaged across these 50 permutations resulting in a time-resolved 96 × 96 neural RDM for each participant (Fig. 2 *A* and *C*). We note here that it is possible to analyze the data using 32 × 32 matrices by averaging across three recordings from each identity such that each cell represents one voice identity. However, we have neither evidence that listeners can reliably perceive the different recordings from the same person as belonging to a single identity (68, 69) nor that listeners perceived the different recordings to be similar in person characteristics (70). As a result, we found that any analyses by identity resulted in overall noisier estimates such that we opted to model the data by stimulus.

For the behavioral ratings, we computed one 96 × 96 behavioral RDM for each of the eight person characteristics based on the absolute differences in average ratings for all 9,216 pairs of voice recordings. For the set of 13 perceptually salient acoustics measures, we conducted a PCA (with oblimin rotation) to reduce the number of dimensions in the data. This PCA yielded 4 principal components with eigenvalues over 1 that explained 78.7% of the variance in the data. For each of these 4 principal components, we computed 96x96 acoustic RDMs based on the absolute differences in principal component scores for each pair of voice recordings. We computed one separate RDM based on the cosine dissimilarity of the LTAS to account for differences in low-level acoustic properties (Fig. 2*C*).

We then computed Spearman’s partial rank correlations between combinations the lower triangles (excluding the diagonal) of these neural, behavioral, and acoustic RDMs. For each person characteristics, we ran one correlation for at each time point at a resolution of 1 ms (from −100 ms to 700 ms relative to stimulus onset.

Specifically, we ran three models (Fig. 2*B*):

• Model 1: The time-resolved Spearman rank correlation between behavioral and neural RDMs partialling out a matrix of the pairwise dissimilarity expressed in cosine distances of the LTAS.
• Model 2: The time-resolved Spearman rank correlation between behavioral and neural RDMs partialling out 1) a matrix of the pairwise dissimilarity of the LTAS and 2) matrices of the absolute differences between 4 principal components characterizing the perceptually salient acoustic properties of the voice recordings. The principal components were derived from a PCA with oblimin rotation including all 13 acoustic measures (see above). The 4 principal components accounted for 78.7% of the variance in the acoustic data.
• Model 3: The time-resolved Spearman rank correlation between behavioral and neural RDMs partialling out 1) a matrix of the pairwise dissimilarity of the LTAS, 2) perceptual-salient acoustics, and 3) all other averaged behavioral matrices partialled out. This model therefore accounts for acoustic and all known perceptual characteristics of the voices.

To establish significance, we used permutation-based cluster-size inference (with null distributions created from 10,000 bootstrapping iterations) as implemented in CoSMoMVPA (71). For one-sample *t* tests against 0, the resulting statistical maps were thresholded at *Z* > 1.64 (i.e., *P* < 0.05, one-sided). For the two-sample *t* tests implemented for comparing timelines across models, the statistical maps were thresholded at Z > 1.96 (i.e., *P* < 0.05, two-sided) as it was unclear what the direction of the effects would be in all cases.

## Supplementary Material

Appendix 01 (PDF)

## Data Availability

Data, stimuli, and scripts for the RSA are available at https://osf.io/n9kvc/?view_only=3a99e1a015104574817700d4f9844242 (72).
