# Supplementary material for: The time course of person perception from voices in the brain
Source: Proc Natl Acad Sci U S A. 2024 Jun 18;121(26):e2318361121. doi: 10.1073/pnas.2318361121 (PMC11214051; doi:10.1073/pnas.2318361121)
Supplement: Supplementary file 1 — Appendix 01 (PDF) [file pnas.2318361121.sapp.pdf]

Supplementary information

Supplementary Analysis 1: Analysis of Auditory Evoked Components

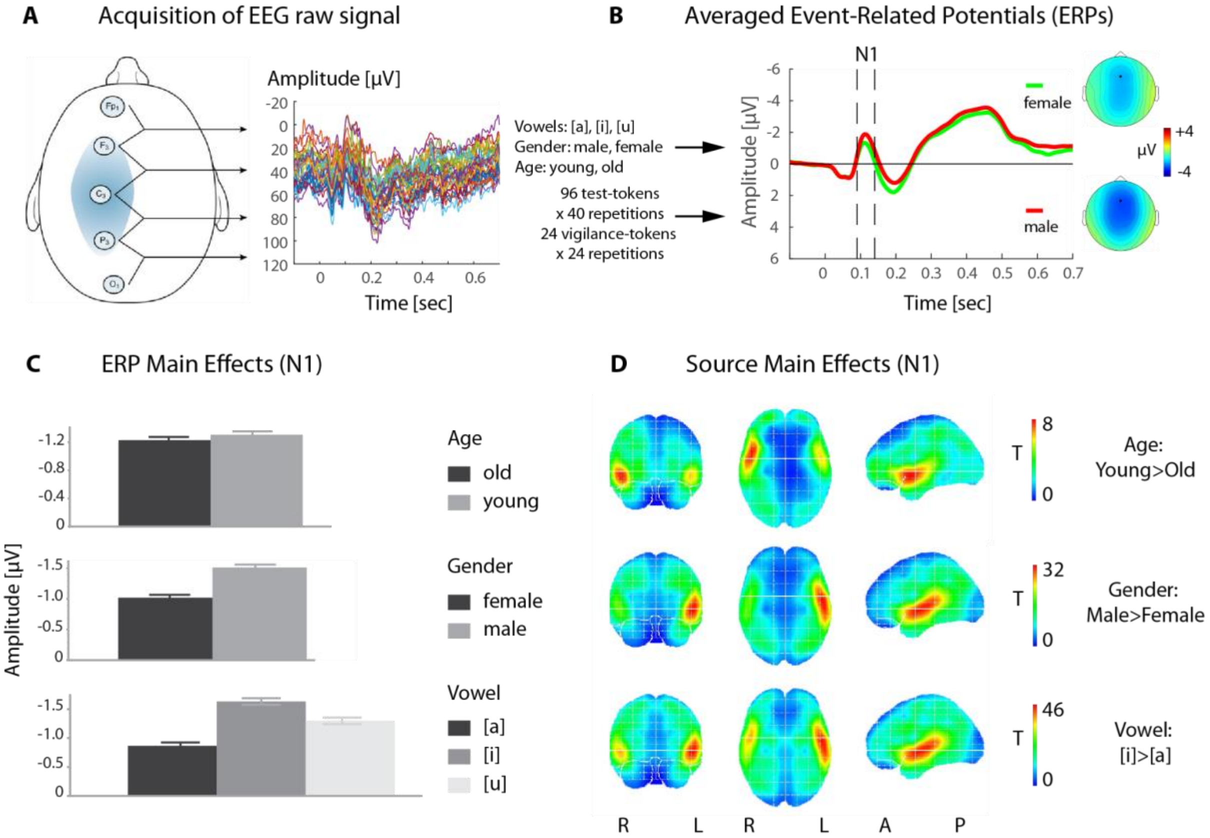

Supplementary Figure 1: Illustration of pre-processing of EEG raw data and analysis of early evoked components. A. The EEG raw signal has been continuously recorded from 32 electrodes (mounted according to the 10-20 convention) and subsequently been epoched with 0.1 sec before and 0.7 sec after stimulus onset. Before averaged across levels of vowel (/a/, /i/, /u/), gender (female, male) and age (young, old), epochs were bandpass-filtered between 0.01 Hz and 30 Hz (using a 4th-order finite impulse response filter as recommended by Widmann et al., 2015), re-referenced against linked mastoids (approximated by the channels TP09 and TP10) and baseline-corrected by subtracting the averaged pre-stimulus epoch (-0.1–0) from each epoch. B. Illustration of a typical averaged event-related potential (ERP), exemplified by averages across levels of gender (female: green, male: red). The first noticeable negative deflection is identified as N1 with its typical fronto-central topography. C. Illustration of the differences in objective between levels of age (young, old), gender (female, male), and vowel (/a/, /i/, /u/) for the stimuli. Note that age and gender effects here are based on the properties of the voices as defined in the Saarbrücker Voice Database and are not based on the same data as the RSA analysis of perceived age and gender in the main text. We also note that the amplitude difference between the vowels /a/ and /i/ has been reported before and aligns with the acoustic-phonetic properties of /a/ (low) and /i/ (high). D. Approximation of cortical sources of the N1 effects (differences between levels of age, gender and vowel). Cortical sources were estimated using the Variable resolution electromagnetic tomography[1]. Differences are color-coded and correspond to T-values. R: right, L: left, A: anterior, P: posterior.

Previous electrophysiological research on speech sound – particularly vowel – processing has focused on auditory evoked potentials (AEPs, i.e., event-related potentials [ERPs] to sounds). In these studies, a prominent AEP is the so-called N1, a negative deflection at around 90-150 ms after stimulus onset with a fronto-central topography[2]. Cortical sources of the N1 have

been reported to lay in bilateral temporal gyri/sulci. Subsequent work has shown that the parameters of the N1 (mean/peak amplitude, latency of peak) are systematically modulated by acoustic-phonetic characteristics of the respective stimuli[3-7] (Eulitz et al., 1995; Mäkelä et al., 2003; Obleser et al., 2003; Roberts et al., 2000; Scharinger et al., 2011). In particular, these studies revealed that information in the lower parts of the frequency spectrum (corresponding to the fundamental frequency, F0, and the first formant frequency of vowels) correlated with N1-amplitude and latency. A robust finding was that lower F1-values (in the vowel /i/ as compared to /a/) corresponded to higher (and later) N1-amplitudes. To allow for comparisons of the current study to existing N1 studies on vowel processing, we here report traditional AEP analyses across the levels of vowel (/a/, /i/, /u/, gender (male, female), and age (old, young)).

To this end, the pre-processed EEG data was averaged across the respective levels of the three factors vowel, gender, and age (Supplementary Figure 1A). Note that the gender and age data in this analysis are the objective measures reported in the demographics of the Saarbrücker Voice Database (i.e., they are the participants' reported age and gender) and are not the same data as the subjective perceptual ratings of gender and age used in the RSA analysis in the main text and Supplementary Analysis 2.

Exemplified by gender, Supplementary Figure 1B shows a typical time course of the AEP, with the first prominent negative peak to reflect the N1 (bound between 90 and 140 ms post-stimulus onset, see dashed lines). Mean N1 amplitudes in the 50-ms-window around the peak were subjected to t-tests and showed significant differences between males and females (t=7.40, d=0.24, p<0.001), with higher N1 amplitudes for male than for female voices. Differences between old and young voices were not significant (t=1.15, d=0.04, p=0.25). Regarding the vowels, /i/ yielded significantly higher N1 amplitudes than /a/ (t=9.36, d=0.37, p<0.001). Similarly, /u/ showed higher N1 amplitudes than /a/ (t=5.34, d=0.21, p<0.001). Finally, /i/ elicited even higher N1 amplitudes than /u/ (t=4.16, d=0.16, p<0.01; for all comparisons, see Supplementary Figure 1C).

We can furthermore approximate a source localisation of the N1 or the N1 effects (i.e., the differences between the levels of the factors gender, age, and vowel). Cortical sources were estimated using variable-resolution electromagnetic tomography (VARETA[1]). This technique tries to reconstruct sources by looking for a discrete spline-interpolated solution to the EEG inverse problem. To achieve this, the VARETA algorithm obtains an estimate of the spatially smoothest intracranial primary current density (PCD) distribution that is compatible with the observed scalp voltage distribution. Possible solutions were restricted to grey matter based on the probabilistic brain tissue maps available from the Montreal Neurological Institute (MNI[8]). The algorithm included the following steps: First, possible sources were modelled as a pre-defined grid of voxels with 7 mm spacing. The 32 electrodes were co-registered with the average probabilistic brain atlas developed at the MNI, assuming a head radius of 85 mm. N1 amplitude differences between the levels of vowel, age and gender were then transformed into source space. Statistical parametric maps (SPMs) of the PCD estimates were constructed based on a voxel-by-voxel Hotelling T2 test against zero (N=32). Higher t-values correspond to warmer colours. The source localisations are illustrated in Supplementary Figure 1D.

Supplementary Analysis 2: Model 0. Decoding person characteristics from the neural data without partialing out any other information

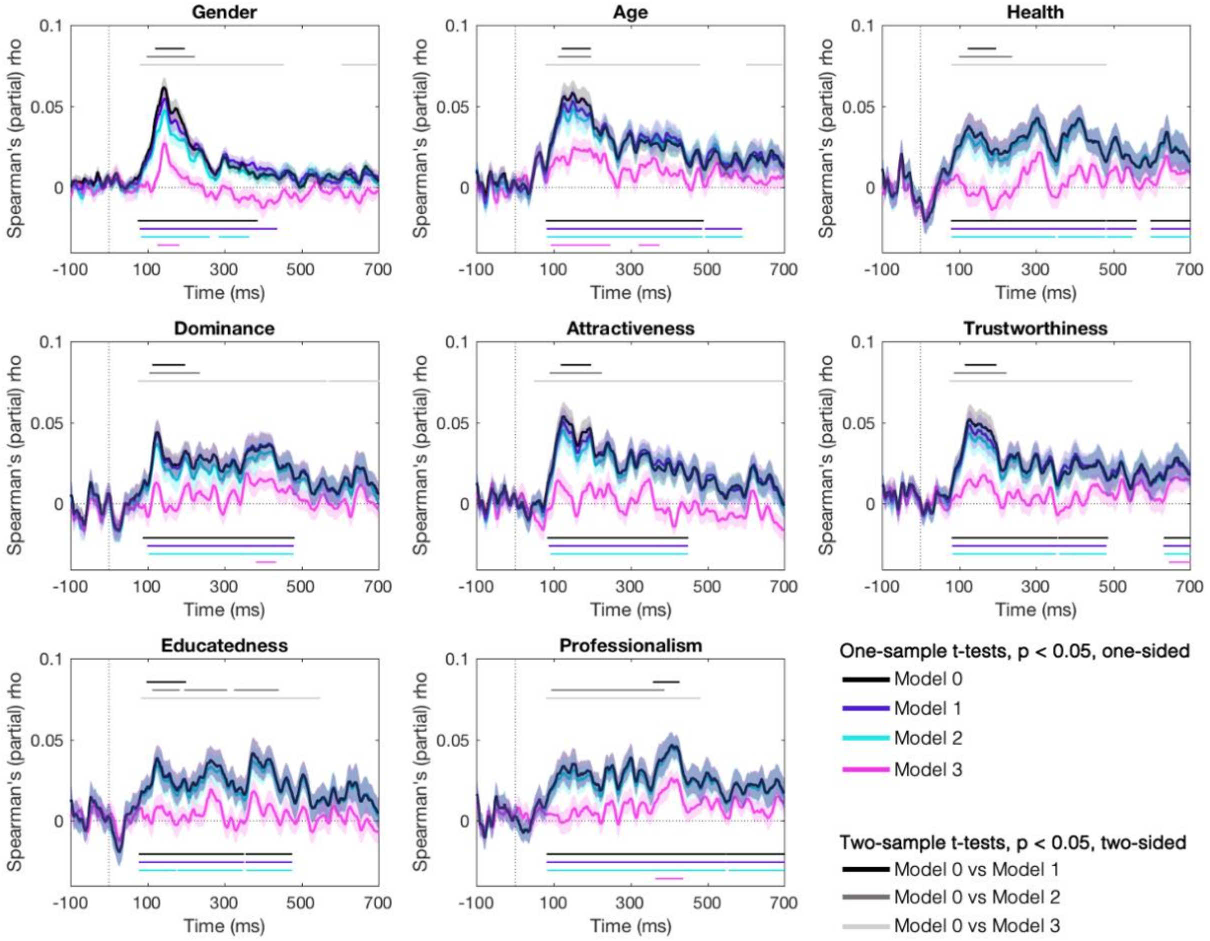

Supplementary Figure 2 Temporal dynamics of person perception from voices, plotted by person characteristics. Black lines show the time courses of person perception derived model Model 0 (no information partialed out, purple lines show time courses of person perception derived from Model 1 (partial correlations between neural and the relevant behavioural matrix, with the LTAS matrix partialed out). Aquamarine lines show time courses of person perception derived from Model 2 (partial correlations between neural and the relevant behavioural matrix, with the LTAS matrix and matrices capturing perceptually salient voice acoustics partialed out). Pink lines show time courses of person perception derived from Model 3 (partial correlations between neural and the relevant behavioural matrix, with the LTAS matrix and matrices capturing perceptually salient voice acoustics, plus matrices of all other known person characteristics partialed out). Shaded areas mark the SEM, significance is marked for p<0.05 (one-sided) for one-sample t-tests and for p<.05 (two-sided) for two-sample t-tests.

In the main text, three different models are reported for the RSA analysis, each looking for increasingly abstracted representations by partialing out different types of information. Here, we additionally represent an analysis including a model where no information has been partialed out (Model 0). As in the main text, we report one-sample t-tests against 0 to establish when representations of the different person characteristics can be decoded from the neural data, as well as two-sample t-tests comparing the different models against each other. While we compared Model 1 against Model 2 and Model 3 in the analysis reported in the main text, we now compare Model 0 against Models 1-3.

Supplementary Figure 2 shows a very similar set of results as reported in the main text. For Model 0, representations for all person characteristics can be found between 90ms and

384ms, with presentations for gender being detectable from 77ms onwards, while representations for health, trustworthiness and professionalism can be detected – with short breaks – up until 700ms after stimulus onset. When comparing Model 1 against Model 0, we can see that the information encoded in the LTAS that has been partialed out in Model 1 to account for low-level acoustic features of the recordings, mainly affects representations during an early time window (exception: trustworthiness). When comparing Model 0 against Model 2, the results are again very similar to the results of the comparison of Model 1 and Model 2, showing that the LTAS and perceptually salient voice acoustics play a role during early voice processing, with some more sustained responses being apparent for educatedness and professionalism. Finally, the comparison of Model 0 and Model 3 also reveals differences between models that stretch across most of the sampled time window in this study.

Supplementary References

1. Bosch-Bayard, J., Valdos-Sosa, P., Virues-Alba, T., Aubert-Vazquez, E., John, E. R., Harmony, T., Riera-Diaz, J., & Trujillo-Barreto, N. 3D statistical parametric mapping of EEG source spectra by means of variable resolution electromagnetic tomography (VARETA). *Clinical Electroencephalography*, **32**(2), 47-61 (2001).
2. Näätänen, R., & Picton, T. The N1 wave of the human electric and magnetic response to sound: A review and an analysis of the component structure. *Psychophysiology*, **24**(4), 375-425 (1987).
3. Eulitz, C., Diesch, E., Pantev, C., Hampson, S., & Elbert, T. (1995). Magnetic and electric brain activity evoked by the processing of tone and vowel stimuli. *Journal of Neuroscience*, **15**(4), 2748-2755 (1995).
4. Mäkelä, A. M., Alku, P., & Tiitinen, H. The auditory N1m reveals the left-hemispheric representation of vowel identity in humans. *Neuroscience Letters*, **353**(2), 111-114 (2003).
5. Obleser, J., Elbert, T., Lahiri, A., & Eulitz, C. Cortical representation of vowels reflects acoustic dissimilarity determined by formant frequencies. *Brain Research. Cognitive Brain Research*, *15*(3), 207-213 (2003).
6. Roberts, T. P. L., Ferrari, P., Stufflebeam, S. M., & Poeppel, D. Latency of the auditory evoked neuromagnetic field components: Stimulus dependence and insights toward perception. *Journal of Clinical Neurophysiology*, **17**(2), 114-129 (2000).
7. Scharinger, M., Poe, S., & Idsardi, W. J. A three-dimensional cortical map of vowel space: Evidence from Turkish. *Journal of Cognitive Neuroscience*, **23**(12), 3972-3982 (2011).
8. Evans, A. C., Collins, D. L., Mills, S. R., Brown, E. D., Kelly, R. L., & Peters, T. M. 3D statistical neuroanatomical models from 305 MRI volumes. Nuclear Science Symposium and Medical Imaging Conference, *IEEE* (1993).
